# Skin and hair-on-a-chip: Hair and skin assembly versus native skin maintenance in a chip-based perfusion system

**DOI:** 10.1186/1753-6561-7-S6-P93

**Published:** 2013-12-04

**Authors:** Ilka Wagner, Beren Atac, Gerd Lindner, Reyk Horland, Matthias Busek, Frank Sonntag, Udo Klotzbach, Alexander Thomas, Roland Lauster, Uwe Marx

**Affiliations:** 1 Technische Universität Berlin - Berlin, Germany; 2 Fraunhofer IWS - Dresden, Germany

## Background and novelty

In recent decades, substantial progress to mimic structures and complex functions of human skin in the form of skin equivalents has been achieved. Different approaches to generate functional skin models were made possible by the use of improved bioreactor technologies and advanced tissue engineering. Although various forms of skin models are successfully being used in clinical applications, in basic research, current systems still lack essential physiological properties for toxicity testing and compound screening (such as for the REACH program) and are not suitable for high-throughput processes.

## Experimental approach

**Figure 1:**
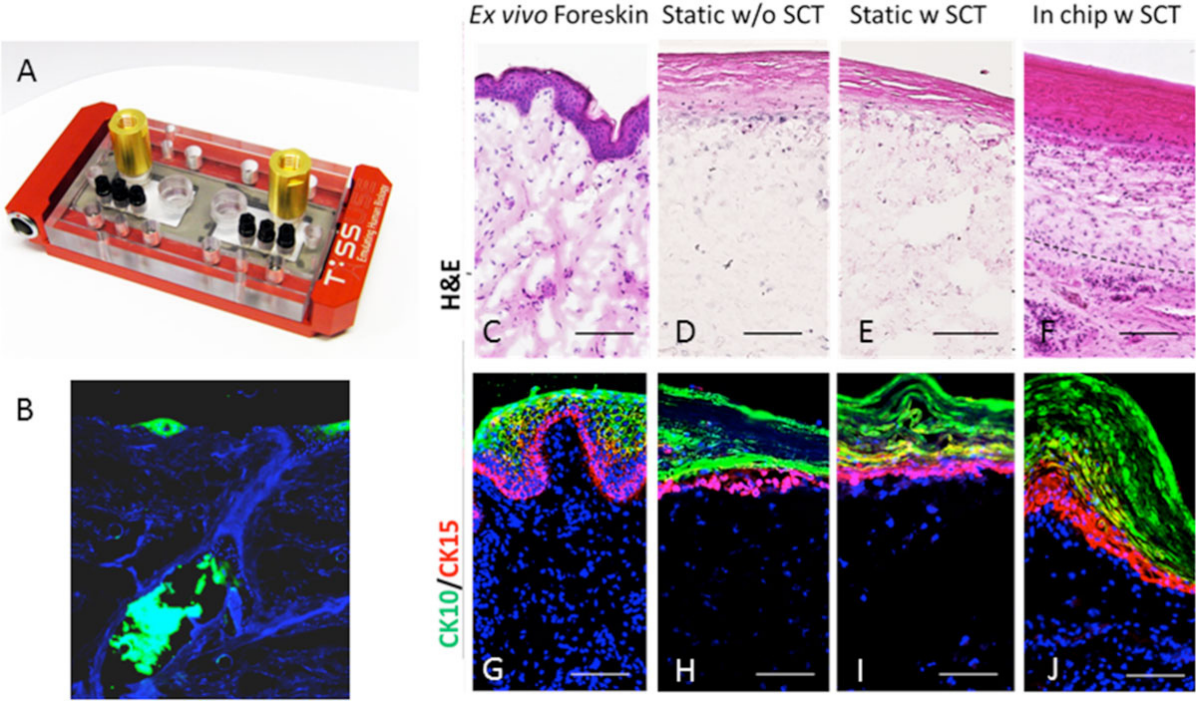
**Microfluidic device for perfused skin equivalent culture and integrated Microfollicle (A) Dynamic chip-based bioreactor for continuous perfusion culture of skin equivalents with integrated microfollicles**. **(B) **PanCytokeratin immunoflourescent staining of a skin equivalent with an inserted microfollicle. (**C-J**) *In vitro *skin equivalents (MatTek) cultured for 7 days in MOC or static conditions with and without subcutaneous tissue (SCT) and compared to *ex vivo *foreskin. **(C-F) **H&E staining and **(G-J) **immunofluorescence staining for epidermal markers Cytokeratin 10 and 15. Dashed lines mark the border between the skin equivalent and the subecuteaneous tissue. Scale bars indicate 100μm.

In particular, further bioengineering is necessary for the implementation of adipose tissue, hair follicles and a functional vascular network into these models. In addition, miniaturization, nutrient and oxygen supply, and online monitoring systems have to be implemented in sophisticated culture systems. To become one step closer to the *in vivo *situation, we produced microfollicles as *in vitro *hair equivalents and integrated them into skin models. These microfollicles containing skin tissues were cultured under static and dynamically perfused conditions and were compared to *ex vivo *scalp and foreskin skin organ cultures. Dynamic cultivation was performed in our Multi-Organ-Chip system (Figure 1 A).

## Results and discussion

The formation of functional neopapillae needs more than 48 hours. After the addition of keratinocytes and melanocytes, the self-organizing microorganoids follow a stringent pattern of follicular-like formation by generating polarized segments, sheath formations and the production of a hair shaft-like fiber. We show that the *de novo *formation of human microfollicles *in vitro *is accompanied by basic hair follicle like characteristics. The microfollicles can be used to study mesenchymal-epithelial-neuroectodermal interactions and for the *in vitro *testing of hair growth-modulating substances and pigmentary effects. As the hair follicle is highly vascularized, it supports penetration of substances into the skin and further into the bloodstream. Testing of topically applied substances might therefore be performed with significantly enhanced validity by the incorporation of a microfollicle into a dynamic chip-based bioreactor containing a skin equivalent which mimics a physiological penetration route. Commercially available skin equivalent EpiDermFT were cultured in the Multi-Organ-Chip for 7 days with subcuteaneous tissue and showed better viability and comparable histological results to native skin (Figure 1 C-J). Cellular and nutritional effect of the subcueaneous tissue is visible even under static conditions. Presence of subcuteaneous tissue decreased the expression of Tenascin C in dermis which is a marker for inflamation and fibrosis. Integritiy of the epidermis and proliferating cells in epidermis kept prominently in combined tissues. Figure 1 B showes the staining of a skin equivalent with an successfully inserted microfollicle.

## Conclusion

Perfusion of the combined tissue provides better integration and associated to viability of the subcuteaneous tissue. In general, presence of subcutaneous tissue increased the longevity of the *in vitro *skin equivalent in both static and especially in Multi-Organ-Chip cultures with improved tissue architecture. A skin equivalent with integrated microfollicles and subcutaneous tissue under dynamic perfusion will be the most suitable model for long-term cultivation and more efficient drug studies and one step closer to mimic *in vivo *skin.

